# Teaching Simulation Literacy with Evacuations: Concept, Technology, and Material for a Novel Approach

**DOI:** 10.1007/978-3-030-57717-9_15

**Published:** 2020-09-07

**Authors:** Andre Greubel, Hans-Stefan Siller, Martin Hennecke

**Affiliations:** 8 Universidad Carlos III de Madrid, Leganés (Madrid), Spain; 9 Tallinn University, Tallinn, Estonia; 10 Open University Netherlands, Heerlen, The Netherlands; 11 Trinity College Dublin, Dublin, Ireland; 12 Graz University of Technology and Know-Center, Graz, Austria; University of Wuerzburg, Wuerzburg, Germany

**Keywords:** Simulation literacy, Evacuation, 21st century skills

## Abstract

As significant policies are based on their expected outcome in computer simulations, literacy of such simulations is necessary for political participation. In this paper, we propose ways to increase such simulation literacy. We discuss simulation literacy from a theoretical perspective and argue for simulating evacuations as a simple and potent topic to increase simulation literacy. Additionally, we present EVA, a novel educational tool to simulate the evacuating of buildings (not only) for classrooms. Lastly, we show different teaching scenarios and exercises for the usage of EVA in an exemplary way. EVA and further teaching material is available online at www.evadid.it.

## Introduction

Computer simulations are powerful tools to predict future events, and exemplary analyze the impact of dependent variables or proposed policies. As notable examples, computer simulations are used to predict the impact of climate change and evaluate different policies like the necessary amount of social distancing in the light of the recent SARS-CoV-2 pandemic. Hence, it is not only possible but reasonable to discuss policies that significantly impact everyday life based on the result of computer simulations. As such, it is desired to teach citizens about computer simulations in order to increase the literacy necessary for political participation in these debates.

With this paper, we contribute to the research questions *What scenarios, tools, and exercises are suitable to increase simulation literacy?* by defining simulation literacy and presenting a technology-enhanced concept for teaching it.

In Sect. 2, we introduce the term *simulation literacy* and argue for its necessity for political participation. In Sect. 3, we break down simulation literacy into smaller, easier teachable skills, and discuss possible domains and properties of exercises for teaching it. In Sect. 4, we present EVA, a novel tool for simulating evacuation scenarios we developed for use (not only) in classrooms. In Sect. 5, we present exercises aimed at increasing simulation literacy (based on EVA) in an exemplary way. Section 6 concludes this paper.

## Simulation Literacy

Up to now, most educational work about simulations and simulation-based learning focused on teaching *with*, rather than *about*, simulations. Despite our best efforts, we could not find a definition of simulation literacy suitable for this concept. Hence, after introducing an established definition for the term simulation, we propose a definition for simulation literacy in this paper.

A *simulation* “is the imitation of a real-world process or system over time. Whether done by hand or on a computer, simulation involves the generation of an artificial history of a system and the observation of that artificial history to draw inferences concerning the operating characteristics of the real system” [1]. A simulation contains a *model* defining the types of objects and *rules* defining their interactions. Objects in the simulation are called *entities*. All entities together form a *scenario* imitating one real-world situation.

Note that, different from *visualizations* and (interactive) *animations*, the scenario of a simulation can be created and changed interactively. Different from (serious) *games*, the interaction focuses on information gain, rather than engagement, and there are no cooperative or competitive aspects like scores for specific (win) states. Unlike most *mathematical models*, simulations have a clear sequence of states whose progression is caused by time. Additionally, simulations are run and observed, rather than solved.

For educational purposes, one can differentiate between *professional* and *educational* simulations. Different from *educational simulations*, the central goal of professional simulations is the creation of new knowledge (rather than teaching about existing knowledge).

### Defining Simulation Literacy

*Simulation literacy* refers to an individuals ability to (i) understand the results of computer simulations, (ii) evaluate significance and limitations of information generated by computer simulations, and (iii) create useful information with computer simulations through the (a) independent usage and (b) critical examination of such simulations.

This definition contains three goals (understand, evaluate, create), two activities (using and examination), and one application area (simulations). Note that simulations literacy focuses on the literacy of professional simulations.^1^

While we use the term *simulation* in a narrow fashion, the skills required for simulation literacy can be understood in an inclusive way. Hence, such skills might be seen as a sub-category of or overlapping with other concepts like 21$^{st}$-century skills [2], critical thinking [4], problem-solving [10] and problem-based learning [14], computational thinking [13], or modeling competencies [7].

Overall, simulation literacy has a clear application area (simulations), narrow goals (understand, evaluate, create), but utilizes and increases a variety of cognitive abilities.

### The Case for Teaching Simulation Literacy

Accepting simulation literacy as a meaningful concept raises the question “Why should I learn it?”. We base our analysis of this question on the assumption of [5]: The educational relevance of a subject is primarily dependent on its contribution to enabling citizens to make decisions in individual or societal regards.

The degree to which citizens are involved in societal decisions may vary. A person might be (i) an expert providing additional information on a subject (ii) a policymaker depending on information provided by experts (iii) A regular citizen voting in a democracy. In each of these roles, simulation literacy is necessary.

First, experts frequently use simulations as “simulations have the advantage that new policies can be explored without disrupting ongoing operations of the real system” [9]. As an example, the results and recommendations in the IPCC climate reports are based on computer simulations [8]. An extensive list of further application areas is provided in [1]. Because of the importance of simulations for creating information, experts frequently need to be simulation literate.

Unlike this, policymakers frequently do not have the knowledge or time to create all the necessary information for a decision. Hence, they rely on experts and advisers to help them. However, they also need to ensure that the information provided by these persons is valid and not just used to manipulate them into a particular decision. As such, while they do not need to be experts themselves, they need to be able to talk to experts, and both understand their findings and evaluate the significance and limitations of them. Hence, if the experts are using simulations, policymakers need to be simulation literate.

A similar argument is valid for regular citizens. In a democracy, citizens should elect policymakers, hold them to account, and participate in (public and private) debates to form a public opinion. To fulfill these responsibilities, they need to understand and evaluate the opinions and decisions of experts and policymakers alike. As such, simulation literacy is required for political participation in (the many) domains where simulations are used to support decision making.

Lastly, citizens need to understand these policies (based on simulations) to accept or decline them and decide how to adapt their behavior accordingly. Note that this is already happening: Simulations and animations of simulations are currently used by the media and academia to spread awareness for the need of social distancing rules in the current SARS-CoV-2 pandemic [11, 12].

Overall, we think that simulation literacy is required for the general education necessary for political participation and personal maturity in the modern world.

However, the necessity of simulation literacy arguably does not imply the necessity of teaching it. Simulation literacy may be automatically acquired by teaching related skills (like problem-solving or modeling competences) in different application areas (like serious games or mathematical models). However, in their list of four disadvantages of simulations, [1] list as first two aspects: “Model building requires special training”, and “Simulation results can be difficult to interpret.” Hence, we think it is unlikely that these skills are picked up without explicit training or as a by-product of teaching focused on a different skill. Either way, further empirical research is necessary to test this assumption.

## A Concept for Teaching Simulation Literacy

Accepting that simulation literacy is a useful concept that should be taught implies the next questions: “How to teach simulation literacy?”. In this section, we (following a normative approach) propose a suitable domain for teaching simulation literacy and a classification system for exercises.

### Teaching Simulation Literacy with Evacuations

As simulation literacy targets methodical competencies (rather than factual knowledge), many domains are suitable for teaching it. In this paper, we argue for and focus on using evacuations of buildings, because this domain has many advantages during teaching: (i) the topic is closely related to everyday experience, (ii) data about scenarios and results are easy to collect (iii) the model and rules are easy to understand, and implementing and evaluating them requires little domain knowledge, (iv) the topic enables many methods and questions.

While being evacuated is not an everyday activity, many everyday experiences revolve around evacuations. For example, almost all public buildings include aerial ladders and escape routes to empty the building quickly. Signs marking these routes have to be clearly visible by law, and their perception is an everyday experience. Additionally, training for real-life evacuations is a periodical activity for many schools, universities, and workplaces. This everyday exposure turns this topic into a motivating learning subject, not only for schools.

These periodical trainings might also act as a useful data source. Both parameters for scenarios (like time required for *x* people to go through a door) and real-world simulation results (like time required to empty a full building) can be collected during these training – notably without ethical implications. As such, little domain or expert knowledge is required to build and evaluate scenarios.

This also is true for the rules of such a simulation, as they primarily revolve around people following a more or less appropriate path to one or more pre-defined secure locations. The people (agents) in this situation do not require a complex internal state as only a few variables influence the behavior – including “my current position” and “utilization of available paths”. Usually, common knowledge is sufficient for categorizing agent behavior as realistic or not.

Lastly, evacuations enable a wide variety of different questions with complexity and difficulty ranging from “identify locations that are prone to congestion in a given building” to “define building regulations necessary to ensure that schools with a given amount of pupils can be evacuated within 10 min”.

Some of these aspects are not all true for other domains. For example, the model and rules of climate simulations are dependent on geological, physical, biological, and chemical models and interactions that are not easily picked up by the average person. Similarly, estimating parameters regarding viral spread (like the impact of wearing face masks against SARS-Cov-2) requires information that, at the time of writing, even current research cannot accurately predict.

However, information regarding evacuations notably is also politically less relevant than corresponding information for viral spread or climate change.

### Classifying Exercises for Teaching Simulation Literacy

Naturally, teaching should neither over- nor underwhelm learners. Hence, exercises should be selected in a way that ensures the suitability of exercises for the current skill level of learners. To support this process, we propose a novel classification system for exercises. In the system proposed, each exercise is classified with a set of *skills* and the scenarios used are classified with a level of *abstractness* and *incompleteness*. In the remainder of this section, we describe the structure and application of such a system in more detail.

**Skills Required for Simulation Literacy.** Our first component, the set of skills required for solving the exercise, regards the goals (understand, evaluate, create) of simulation literacy. These goals themselves are commonly understood as skills of increasing difficulty [3, 6]. As they additionally influence the approach for solving an exercise, we include them in our model. To enable a more precise categorization, we break them down into smaller, more directly teachable skills.

To understanding results, students need to:

1. apply domain knowledge or common sense to translate observations about the simulation into claims about the real world.
2. apply descriptive statistics to translate numbers generated by the simulation into claims about the real world.

To evaluate significance and limitations, students need to:

1. apply domain knowledge or common sense to identify deviations between the simulation and the real world.
2. identify the cause (e.g., model, scenario, ...) for these deviations.
3. identify different goals of the simulation (e.g., low resource demand, simple design, realistic representation of the world, ...).
4. classify pairs of simulation goals into the categories “dependent on each other”, “independent from each other”, and “mutually exclusive”.
5. use variance analysis, experimentation, or critical reasoning to quantify the impact of deviations and competing goals.
6. evaluate the impact of deviations and competing goals onto the generated claims about the real world.

To create information, students need to:

1. create new information by adapting existing scenarios.
2. translate real-world situations into new scenarios for a given simulation.
3. implement new models and rules for a simulation.

Unfortunately, C3 can require a variety of different material and skills like computing resources, or programming and project management skills. Furthermore, it is very time consuming to implement (i.e., program) a computer simulation from scratch. In practice, it probably is necessary to use existing simulations with pre-defined models and rules and only implement new scenarios with an editor. In fact, we base the remainder of our paper on a simulation tool we developed specifically to mitigate this problem. However, teachers must ensure that the model and the rules underlying a pre-existing simulation are properly discussed and verified. It is a strong necessity to fight the misconception “Every simulation is valid and useful if one creates the right scenario” as it directly contradicts the goal of evaluating the significance and limitations of simulations.

**Level of Abstractness.** Additionally to the set of skills, the difficulty of an exercise also depends on the level of abstractness. The level of abstractness describes how many different scenarios need to be considered for solving this exercise. This consideration can be implicit if a single scenario solves the exercise, but finding it requires consideration of multiple scenarios. The difficulty of an exercise increases with the abstractness of a scenario. An exercise can be:

- concrete: It uses a single real-world scenario in a given tool.
- representative: It uses a few real-world scenarios in a given tool.
- generic: It uses multiple real-world scenarios in a given tool.
- tool-based: It uses multiple edge cases and scenarios not representing the real-world in a given tool.
- model-based: It uses multiple scenarios with a given model
- abstract: It uses multiple scenarios with different models in a given domain

**Level of Incompleteness.** As the last dimension, we use the level of incompleteness that describes the amount of steps (like implementing entities or changing parameters of entities) necessary to execute a scenario. The difficulty of an exercise increases with the level of incompleteness. A scenario can be:

- finished: All entities are implemented and have reasonable parameters.
- mostly finished: All entities are implemented, most have reas. parameters.
- unfinished: Some entities are missing, but most existing entities have reasonable parameters.
- started: Most entities are either missing or have unreasonable parameters.
- defined: No entities are implemented, but they are described in detail (e.g., by a floor layout with markers for waypoints and persons).
- blueprinted: No entities are implemented, but the real-world situation is described in detail (e.g., by a floor layout).
- described: No entities are implemented, and the real-world situation is circumscribed (e.g., by providing a verbal description of a building).

To create a right difficulty curve during learning, we apply these categories like a spiral in our material (cf. Sect. 5): We start with exercises focused on simple skills and low level of abstractness and incompleteness.^2^ Then, similar to a spiral curriculum, we increase difficulty in one dimension and rotate the dimension of difficulty increase over time.^3^

**Fig. 1.**
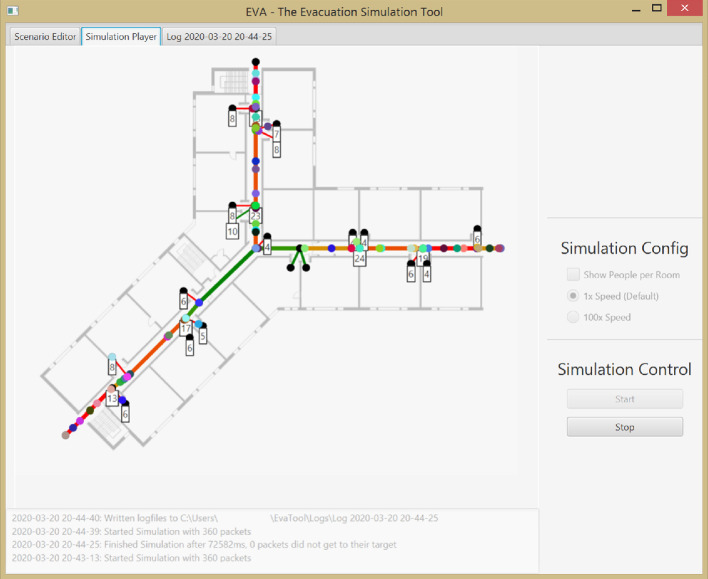
Snapshot of EVA during the execution of a evacuation simulation.

## Technology for Teaching Simulation Literacy: EVA

As discusses before (cf. Sect. 3), it is impracticable to create computer simulations in school from scratch. In this section, we present EVA, a novel educational tool we developed for teaching simulation literacy. EVA is developed in Scala and can simulate the evacuations of buildings by providing three core functionalities:

1. Implement evacuation scenarios using a graph-based flow network as a model.
2. Execute the implemented scenario using agent-based rules.
3. Analyze statistical information about the executed simulation.

We will describe these functionalities in more detail in the next section.

The user interface (depicted in Fig. 1) consists of three areas: The top-left MainArea provides the core functionalities in three tabs that show the graph of the flow-network. Depending on the current tab, this flow-network is overlayed with different information like the current state of an active simulation. Additionally, the right ControlArea allows the change of properties in the current tab (e.g., increasing the number of persons at a selected node or stopping the simulation). Lastly, the bottom LogArea logs essential events.

**Fig. 2.**
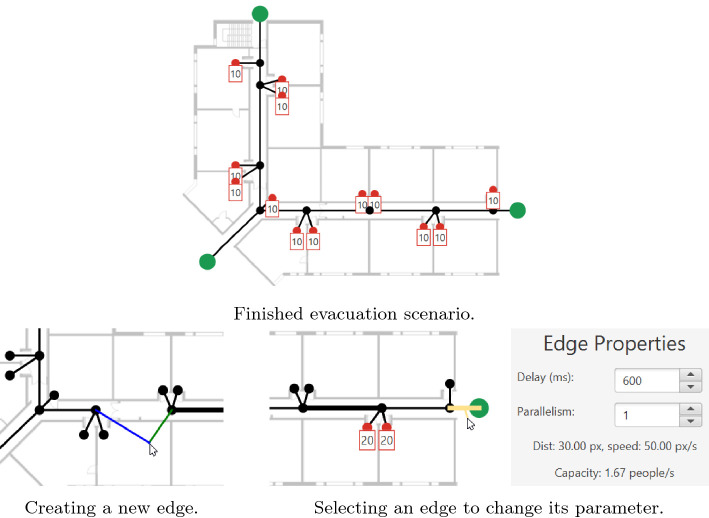
Gui details of the scenario editor in EVA (cropped snapshots).

### Implement Evacuation Scenarios

The evacuation scenario uses a graph-based flow network as a model. Edges represent escape routes in the model and are bordered by nodes depicting rooms or waypoints. A node also can be a safe point acting as a destination for the escape paths. Every room may contain a certain number of initial persons trying to get to these safe points. Edges can be configured to change the duration (in ms) it takes to get from one to the other end. Additionally, the amount of people simultaneously allowed on one edge can be changed.

In the *Scenario Editor*, nodes can be added by clicking on the desired position in the editor. Right-clicking removes the nearest node. A background image of the floor layout can be added to guide this creation process. By changing the editor mode to “Add Edges”, edges can be added (removed) by left (right) clicking on the desired nodes. The last editor mode, “Change Parameter”, allows for the selection of nodes and edges to change their parameters. These parameters also influence the color, size, and annotation of nodes and edges.

Figure 2 shows various snapshots of the editor during the creation of a scenario, as well as a finished evacuation scenario where every room contains ten persons trying to flee to one of the three green safe points outside the building.

### Execute Evacuation Simulations

The modeled scenarios can be executed in the *Simulation Player*. Figure 1 shows a snapshot during an execution. The colored, moving circles on the edges picture fleeing persons. Numbers annotated on the nodes denote the number of persons currently waiting at this node for an edge to become available. The current utilization of the edge, defined as the ratio between the current and maximum amount of persons on this edge, determines the color of the edge, ranging from green (empty edges) to red (full edges).

**Agent Behavior.** During the simulation, every agent (person) is trying to get to one of the safe nodes. The following algorithm determines the next edge to take for an agent:^4^ 
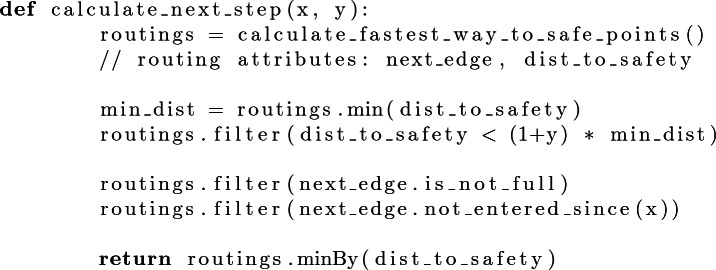
 If this algorithm returns an edge, the agent will take it. Otherwise, the agent remains at the current node. If an agent leaves an edge, every agent at the bordering nodes invokes this algorithm again in order of arrival.^5^

**Parameter Choices.** The parameter x is introduced for the human readability of the simulation. If choosing x = 0 ms, multiple people would enter an edge (almost) simultaneously. Hence, the corresponding circles are (almost) at the same position, reducing the readability of the simulation. We currently use x = 50ms to limit the impact of this effect.^6^

The parameter *y* is introduced to enable agents to distribute themselves to different paths, even though one is longer. As an initial configuration, y = 40% is chosen. While this choice leads to realistically seaming results in standard configuration, it is deliberately arbitrary and can and should be discussed with learners (c.f. Sect. 5).

### Analyze Simulation Results

During the simulation, statistical information is automatically gathered. After the simulation finishes, key figures are shown in the evaluation tab. The MainArea displays an image of the graph displaying the amount of persons that visited each node and the (color-coded) utilization for each edge.

A table in the ControlArea also lists the following information: (i) the time the simulation started, (ii) the time the simulation finished, (iii) the duration of the simulation, (iv) the number of persons that successfully fled to save points, and (v) the sum of the waiting duration of all persons during the simulation.

Clicking on a node displays the following additional information: (i) The number of persons that visited that node, (ii) the sum of the waiting duration of persons waiting at this node for an edge to become available, (iii) the maximum number of persons simultaneously waiting at this node.

Clicking on an edge displays: (i) The number of persons that traveled on this edge, (ii) the sum of the waiting duration of persons that waited for this edge to become available, (iii) the utilization of this edge,^7^ (iv) the duration this edge was active,^8^ and (v) the active utilization of this edge.^9^

Lastly, multiple *person logs* are created that contain detailed information about when which person arrived at which location. They can be used for further analysis and are created for (i) all events, (ii) all events at a specified edge, (iii) all events at a specified node, (iii) all events regarding a specified package.

**Fig. 3.**
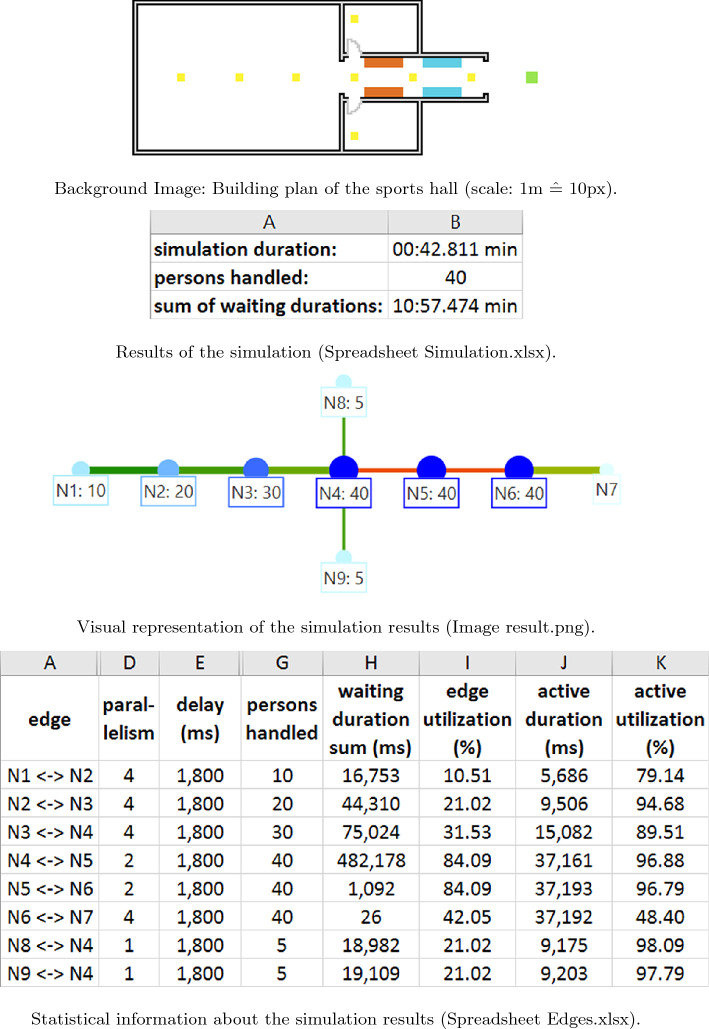
Material for the exercise Sports Hall.

This information is automatically stored to four Excel spreadsheets and one image. Excerpts from these files are shown in Appendix A, Fig. 3. A button in the ControlArea can open the directory containing these files in the file explorer.

## Material for Teaching Simulation Literacy

In this section, we present material (based on EVA) for teaching simulation literacy in an exemplary way. This material is based on the approach elaborated earlier and intended for usage in the last years of K-12 STEM education. The introduced classification system (cf. Sect. 3) is used to classify the tasks.^10^ However, due to spacing restrictions, only one exercise can be provided in detail.^11^ Otherwise, only the underlying ideas are presented. Further exercises, material, solutions, and EVA itself are available online at www.evadid.it.

### Sample Exercise: Sports Hall

The first exercise regards the evacuation of a sports hall. The task description and information necessary for solving this exercise (otherwise generated by the simulation tool) is provided in Appendix A. This exercise is used as one of the first exercises after students got familiar with the basic interaction with EVA.

The tasks A.1–A.3 focus on the translation process between the real-world and simulation results. Because of this, the scenario deliberately uses parameters that are internally consistent (distances are measured in *px* and speed in *px*/*s*) but do not reflect the real world. While working on these exercises, the primary activity of learners is using the simulation tool and interpreting its results.

**Skills**: U1, U2 $\bullet$ **LoA**: Concrete $\bullet$ **LoI:** Finished

This is in contrast to the following tasks (A.4–A.7) that require changing the scenario and examining the results. However, it is not yet necessary to abstract from the scenario given.

**Skills**: E1, E5, E6 $\bullet$ **LoA**: Concrete $\bullet$ **LoI:** Mostly Finished

### Exercise Blueprint: Working with Incomplete Scenarios

As a more complex exercise, learners can use EVA to generate usable data. A sample exercise could be structured as follows: Students are presented with a floor layout and graph in which the nodes and edges in the hallways already exist. The task is to verify the sensibility of the existing plan and finish it.

A first sensible shortcoming to identify is lacking internal consistency between the delays of the edges. In the sports hall example, all edges have the same length in pixels and the same delay. Each edge can have one person simultaneously on it for every 200 cm * 50 cm empty rectangle on the floor (up to a total of four). A next exercise might use edges that are not coextensive in length but have the same delay. Afterward, the learner should finish the scenario by improving the existing steps and adding the edges from the rooms to the hallways. Experimenting with peers can be used to find missing parameters like the time it takes for people to leave through a classroom door.

**Skills**: C1, E1, E5, E6 $\bullet$ **LoA**: Concrete $\bullet$ **LoI:** Unfinished

Another task might additionally present several finished scenarios. Learners then should evaluate which scenario imitates the real-world situation best.

**Skills**: E1, E5, E6 $\bullet$ **LoA**: Representative $\bullet$ **LoI:** Finished

### Exercise Blueprint: Answer Questions and Debate Objections

Evaluation exercises can be stated as objections to the simulation or questions regarding its validity, like tasks 5–7 in the sports hall example. Further reasonable objections and questions include:

(i) In the simulation, people move in groups, rather than equidistant.
(ii) Nodes can contain arbitrarily many persons waiting at them.
(iii) A fire makes certain ways impassable. How can this be considered?
(vi) How to construct a scenario that leads to unrealistic agent behavior for a given value of the parameter *y*?
(v) How to determine the time needed to evacuate a given school?
(vi) How accurate can evacuations be simulated using a graph-based model?

These exercises are increasing in their level of abstractness and incompleteness.

## Conclusion and Future Work

In this paper, we introduced the concept of simulation literacy and presented a technology-enhanced approach for teaching it. EVA and teaching material is available online at www.evadid.it. A translation of the user interface and teaching material into additional languages currently is in development.

As simulation literacy is a concept that previously achieved very little attention, we hope that our definition and this paper offers a new perspective on the set of skills necessary for the 21st century. Additionally, we hope that the concept, tools, and material we presented prove useful for practitioners.

However, research on simulation literacy is far from finished. Notably, we only presented a single approach. Other approaches might be just as feasible (or even better) than our approach. As such, we welcome discussion on further approaches. In our next steps, we want to perform an empirical study analyzing the effectiveness of EVA. More precise, we are interested in how learners interact with EVA, what aspects they focus on during their critical examination, and what skills they can transfer to simulations using different tools or domains.

## A Sample Exercise: Improving the Sports Hall

This task targets students in the last years of their K-12 STEM education at the beginning of their learning, but after they familiarized themselves with the basic interaction with EVA.

### A.1 Task Description

The building department wants to analyze how fast a school can be evacuated. As part of this analysis, they created a building plan of the sports hall which contains a main room, two changing rooms, and a hallway with four lockers. They built the model using the scale 10 px per 1 m and created it as a scenario for their simulation tool.

1. Open and execute the scenario “sports hall”. How long does it run?
2. In the simulation, every person moves with 50 px/s. Calculate the real time it takes to evacuate the building if people are instead moving with 6 km/h.
3. Verify the solution by simulating the scenario with adjusted edge delays.

The building department orders the school to remove half their lockers in the hallways to speed up the building’s evacuation. However, the school board disagrees on the lockers to remove:

(A) We should remove the blue lockers as people can flee faster if the exit is free.
(B) We should remove the red lockers as most people waited for this segment.
(C) We should remove one red and one blue locker as this broadens the hallway.

Removing one locker allows for one additional person to use the corresponding segment of the hallway.

4. Simulate the impact of the proposals on the evacuation time.
5. Argue why some proposals do not speed up the evacuation by much.

After these results, the school board decides to remove two of the lockers. However, not everyone believes this was necessary. Some members argue that the simulation is unrealistic and not suitable to answer this question:

(A) The results are not plausible: How can people wait for over 10 min if the simulation only lasts for less than a minute?
(B) The simulation is unrealistic: Not all people move with 6 km/h. Instead, different people move at different speeds, ranging from 5–7 km/h.
(C) I re-build the scenario and got a different result. It now takes 43 instead of 42 s. If the simulation does return different values after re-building the scenario, it is broken and should not be used!

Analyze these arguments and take a stand on them:

6. Argue whether these arguments are correct or not.
7. Analyze for every correct argument, how it influences the simulation.

## Footnotes

1 However, this does not imply that one cannot use educational simulations to teach literacy of professional simulations.

2 Note that the dimensions we just defined focus on aspects that are specific to simulations. Additional, more general dimensions (like the amount of possible answers) complement the dimensions we propose. Such dimensions are explored in [15].

3 Note that we rotate aspects within a topic, rather than topics within a subject.

4 As common, filter denotes a function that keeps elements in the list that *satisfy* the passed equation (i.e., the equation evaluates to true for the elements retained).

5 Hence, the longest waiting agent will have the first possibility to move on.

6 If using default parameter, i.e. $agent\_radius=5$ px, $edge\_delay=edge.dist/50$ ms, and $x = 50$ ms, every person is at most half overlapped with other persons. We consider this to be sufficient to visible differentiate multiple agents in practice.

7 Defined as: $$persons\_handled/(capacity\_per\_s * simulation\_time)$$.

8 Defined as: $$time\_last\_person\_left\_edge - time\_first\_person\_entered\_edge$$.

9 Defined as: $$persons\_handled/(capacity\_per\_s * active\_duration)$$.

10 We abbreviate *Level of Abstractness* as *LoA* and *Level of Incompleteness* as *LoI*.

11 Learning simulation literacy requires more exercises than the few presented here.
